# Silencing of Barkor/ATG14 sensitizes osteosarcoma cells to cisplatin-induced apoptosis

**DOI:** 10.3892/ijmm.2013.1578

**Published:** 2013-12-09

**Authors:** ZHIFANG ZHAO, LIJIANG TAO, CHENGCHUN SHEN, BING LIU, ZHENGMING YANG, HUIMIN TAO

**Affiliations:** 1 Department of Orthopedic Surgery, The Second Affiliated Hospital, School of Medicine, Zhejiang University, Hangzhou, Zhejiang, P.R. China; 2 Department of Orthopedic Surgery, The 117th Hospital of People’s Liberation Army, Hangzhou, Zhejiang, P.R. China

**Keywords:** osteosarcoma, Barkor/ATG14, caspase-12, apoptosis, cisplatin

## Abstract

Although surgical excision following neoadjuvant chemotherapy has contributed to the long-term survival of osteosarcoma patients, patients that do not respond to commonly used drugs including cisplatin, have a poor prognosis. Autophagy is important in the inhibition of chemotherapeutic apoptosis. Therefore, we investigated whether knockdown of Beclin1-associated autophagy-related key regulator (Barkor/ATG14) promoted cisplatin-induced apoptosis in a drug-resistant osteosarcoma cell line *in vitro*. Saos-2 cells were transfected with Barkor siRNA. Sensitivity of the Barkor siRNA-transfected cell line to cisplatin was evaluated. Silencing of Barkor did not directly inhibit the growth rate of the transfected cells, but it significantly increased their sensitivity to cisplatin. The results of flow cytometry and 4′,6-diamidino-2-phenylindole (DAPI) staining revealed that Barkor siRNA-transfected Saos-2 cells treated with cisplatin exhibited much higher rates of apoptosis than the control and control siRNA-transfected cells. Additionally, the combination of silencing of Barkor with cisplatin treatment promoted the expression of caspase-12 and calpain. The increase of cisplatin cytotoxicity may therefore be involved in endoplasmic reticulum (ER) stress-associated apoptosis. Bcl-2 was markedly downregulated in dose-dependent cisplatin-treated Barkor-transfected-Saos-2 cells. Findings of the present study suggest that the combination of silencing of Barkor and cisplatin enhanced the antitumor efficacy through the Barkor-related ER- and mitochondrial-mediated apoptotic pathway.

## Introduction

Primary osteosarcoma is the most common bone tumor occurring in childhood and adolescence, comprising 2.4% of all malignancies in pediatric patients (1,2). Although neoadjuvant chemotherapy followed by surgical excision has ameliorated the long-term survival of osteosarcoma patients, patients that do not respond to commonly used drugs such as cisplatin, doxorubicin and methotrexate have a poor prognosis (3,4). The identification of the critical molecules and/or signal transduction pathways responsible for regulating development to drug resistance is therefore significant for the development of novel treatment strategies for this type of cancer.

Autophagy, a catabolic process for the autophagosomic-lysosomal degradation of cytoplasmic contents, is characterized by the formation of autophagosomes (double-membrane vesicles) (5,6). Autophagy is associated with a number of physiological processes including differentiation, neurodegeneration, infection, and cancer (7). Findings of recent studies have demonstrated that autophagy protects cancer cells from drug-induced apoptosis and facilitates development to drug resistance (8,9). Autophagy-related (Atg) genes are involved in the formation of autophagosomes, which are delivered to lysosomes for degradation. Atg14, also known as Beclin1-associated autophagy-related key regulator (Barkor), localizes to autophagosomes, isolation membranes, and endoplasmic reticulum (ER) and capable of enhancing Vps34 activity. Knockdown of Barkor inhibits starvation-induced autophagy (10,11). Furthermore, Barkor recruits a series of class III PI3-kinase to the ER, where otherwise phosphatidylinositol 3-phosphate (PI3P) is essentially absent. The Barkor-dependent appearance of PI3P makes ER the platform for autophagosome formation.

The ER-resident caspase-12 has been found to mediate apoptosis signaling induced by ER stress (12). An initial study on caspase-12 knockout mice showed increased resistance to ER stress-induced apoptosis (13). Another protease, caspase-7, is also involved in the activation of caspase-12 in response to ER stress and has been reported to translocate from the cytosol to the ER to interact with caspase-12 leading to its activation (14–17).

The aim of the present study was to determine whether knockdown of Barkor is crucial in osteosarcoma cell chemosensitivity to cisplatin-induced apoptosis through the activation of ER stress-associated apoptosis.

## Materials and methods

### Cell culture

The established human Saos-2 osteosarcoma cell line (HTB-85™, ATCC) was supplied by the Cell Bank of the Shanghai Institute of Biochemistry and Cell Biology, Chinese Academy of Sciences (Shanghai, China). The Saos-2 cell line was cultured for <3 months in McCoy’s 5A medium supplemented with 1% penicillin/streptomycin and 15% fetal bovine serum (Gibco, Grand Island, NY, USA) at 37°C, 5% CO_2_.

### Reagents and antibodies

Cisplatin (diluted in anhydrous DMF) (P4394) was purchased from Sigma Chemical Company (St. Louis, MO, USA). The enhanced chemiluminescence (ECL) kit was from Thermo Scientific Pierce (no. 32109; Rockford, IL, USA). The Barkor and control siRNA were purchased from Cell Signaling Technology (Danvers, MA, USA). Barkor, cleaved PARP, cleaved caspase-9, Bcl-2, Bcl-xl, phospho-p38 MAPK and calpain antibodies were purchased from Cell Signaling Technology, and the Annexin V-FITC/propidium iodine (PI) apoptosis detection kit was purchased from Biouniquer Technology, Nanjing, China.

### CCK-8 viability assay

Saos-2 cells were seeded at 1×10^4^ cells/well in a 96-well plate. The cells were treated for 48 h and incubated for an additional 60 min at 37°C in 10% CCK-8 dye (Dojindo, CK04). In the case of CCK-8 assay, water-soluble tetrazolium salt (WST-8) was reduced by dehydrogenases in cells to yield an orange-colored product (formosan), which is soluble in the tissue culture medium. The amount of the formazan dye generated by dehydrogenases in cells was directly proportional to the number of viable cells. The absorbance was measured at 450 nm using a microplate reader (Bio-Rad 550, Hercules, CA, USA).

### siRNA knockdown of Barkor

The human small interference RNA was used to inhibit mammalian Barkor/ATG14. Cells were transfected with a pre-designed siRNA (100 nM) against Barkor (Cell Signaling Technology, 6286) using the Lipofectamine™ 2000, according to the manufacturer’s instructions. To assay the downregulation effect on Barkor, the expression of protein was detected through western blot and Quantitative real-time polymerase chain reaction (qRT-PCR) following transfection with siRNA for 48 h. During the period of maximal protein knockdown, the cells were treated with cisplatin following siRNA-Barkor transfection.

### Flow cytometry: Quantification of apoptosis

To detect apoptosis, floating cells in the medium and adherent cells were collected after 48 h of treatment. The cells were stained using an Annexin V-FITC/PI Apoptosis Detection Kit (Biouniquer), according to the manufacturer’s instructions. Untreated cells were used as the control. The samples were analyzed using a FACSCalibur flow cytometer (Becton-Dickinson, North Ryde, New South Wales, Australia) within 45 min after the staining.

### Analysis of apoptosis by DAPI staining with laser confocal fluorescence microscopy

DNA damage characteristic of apoptosis was identified by staining with 4′,6-diamidino-2-phenylindole (DAPI). Osteosarcoma cells treated with GA for 48 h were washed three times in phosphate-buffered saline (PBS), and fixed in 4.0% paraformaldehyde at room temperature for 30 min. Samples were stained with 1 μg/ml of DAPI (Sigma Chemical Company) for 15 min, rinsed, and analyzed by laser confocal fluorescence microscopy. Apoptotic cells were characterized by the decrease of nuclear chromatin, fragmentation, or margination to the nuclear membrane.

### Western blot analysis

Total cellular protein extracts were prepared by scraping the cells into Mammalian Protein Extraction Reagent (Thermo Scientific Pierce, no. 78503). Protein concentration was measured using BCA protein assay (Thermo Scientific Pierce). The protein samples were separated on SDS-PAGE at 10–15%, and electrotransferred onto nitrocellulose membranes (Amersham Pharmacia Biotech, Zurich, Switzerland). The membranes were blocked by 5% non-fat milk in Tris-buffered saline Tween-20 (TBST, pH 7.6) for 60 min at room temperature. Primary antibodies (goat anti-mouse IgG-HRP; Thermo Scientific Pierce) diluted in 5% BSA of TBST were incubated overnight at 4°C. The membranes were incubated in secondary antibodies (goat anti-rabbit IgG-HRP; Thermo Scientific Pierce) for 90 min at room temperature. The membranes were washed three times using the above-mentioned procedures. Proteins were detected using electrochemiluminescence (ECL, Amersham Pharmacia Biotech).

### Gene expression detected by qRT-PCR

Total RNA was prepared using TRIzol reagent (Invitrogen, Carlsbad, CA, USA) according to the manufacturer’s instructions. Total RNA (2 μg) was used to synthesize the first strand of cDNA. qRT-PCR was performed using Applied Biosystems 7500 Real-Time PCR System and SYBR^®^ Premix Ex Taq™kit (Perfect Real Time) (Takara Bio Inc., Shiga, Japan). β-actin was applied as the input reference. Results are presented as CT values, defined as the threshold PCR cycle number at which an amplified product is first detected. The CT was determined as the mean of the triplicate CT values for target gene minus the mean of the triplicate CT values for β-actin. The primers used were: Barkor, forward 5′-CACGCCTGTAATCCCAGCTACTC-3′, and reverse 5′-GCAATGGCACAATCTCGGCTCACT-3′; 18S rRNA, forward 5′-GACTCAACACGGGAAACCTCAC-3′, and reverse 5′-CCAGACAAATCGCTCCACCAAC-3′.

### Statistical analysis

Data are expressed as mean ± standard deviation. The mean values were calculated from data obtained in triplicate from each experiment. The statistical significance of differences was determined by Student’s two-tailed t-test in two groups and one-way ANOVA in multiple groups. The data were analyzed using SPSS 17.0. P-values were two-tailed and P<0.05 was considered statistically significant. Asterisks indicate the level of significance.

## Results

### Upregulated expression of Barkor in cisplatin-treated Saos-2 cells

**Figure 1:**
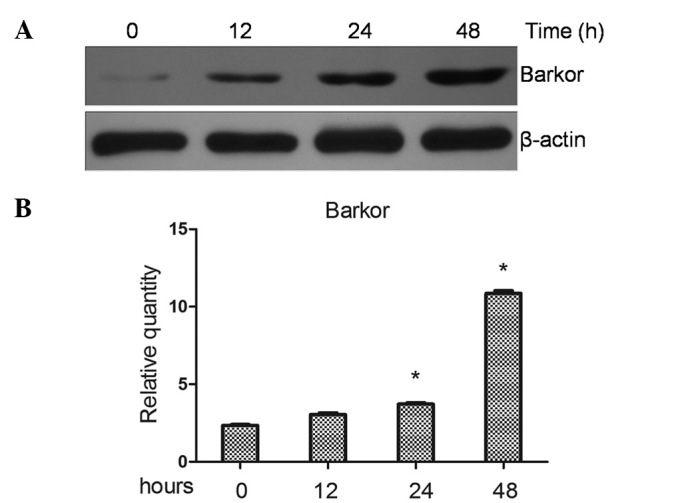
Cisplatin enhanced the expression of Barkor mRNA and protein levels in Saos-2 cells. Cells were treated with cisplatin (10 μM) for 12, 24 and 48 h and prepared for (A) western blotting and (B) qRT-PCR. Quantification of band intensity in (B) normalized to that of mRNA (relative quantity) is shown (n=6). Bars show the average intensity (relative quantitiy) of each band ± standard deviation (^*^P<0.05). Barkor/ATG14, Beclin1-associated autophagy-related key regulator; qRT-PCR, quantitative real-time polymerase chain reaction.

Following exposure to apoptotic stimuli, Saos-2 cells may activate survival mechanisms to evade the induction of cell death. It has been reported (8) that autophagy protects cancer cell from chemotherapeutic drug-induced apoptosis, and Barkor is a key regulator of autophagy. In this study, the Saos-2 cells were treated with cisplatin and Barkor protein levels were observed by western blotting. Results of the western blotting revealed that cisplatin increased Barkor protein levels of the Saos-2 cell line (Fig. 1A). By contrast, qRT-PCR was used to investigate the effects of cisplatin on Barkor at the transcriptional levels. Cisplatin significantly increased the mRNA transcripts of Barkor in Saos-2 cell lines (P>0.05) (Fig. 1B).

### Barkor siRNA transfection did not inhibit Saos-2 cell growth

**Figure 2:**
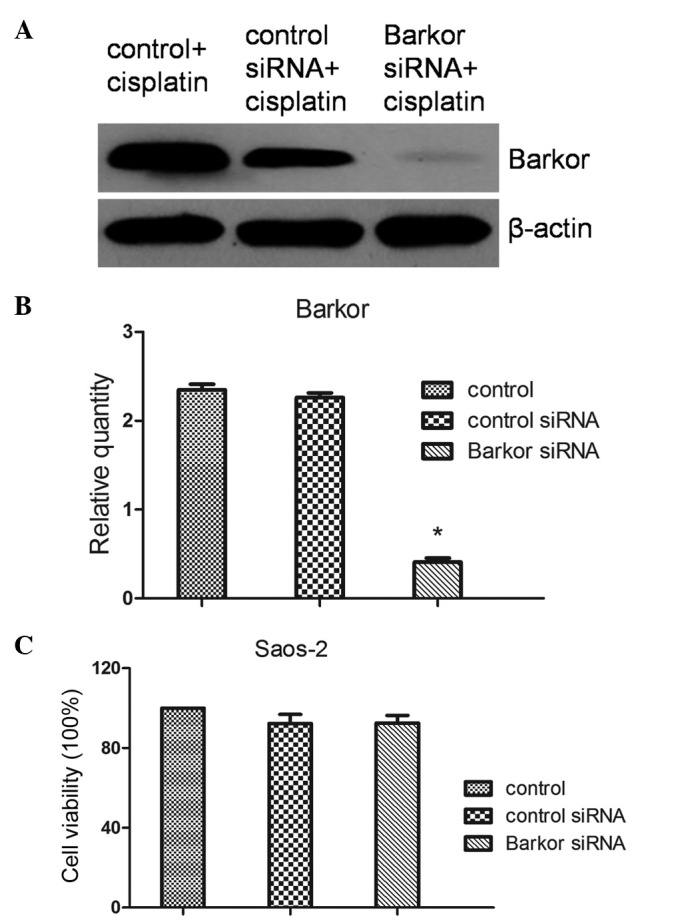
The effect of Barkor siRNA transfection on Saos-2 cell growth. Saos-2 cells were transfected with Barkor- or control-siRNA for 48 h, after which the Barkor expression level was analyzed by (A) western blotting and (B) qRT-PCR. Bars show the average intensity (relative quantity) of each band ± standard deviation (n=6) (^*^P<0.05). (C) The CCK-8 assay was used to measure the growth rates in Barkor-siRNA- and control-siRNA-transfected Saos-2 cells after transfection for 48 h. The proliferation rates were similar in the Barkor-siRNA- and control-siRNA-transfected and control cells. Bars show the average intensity of each band ± standard deviation (n=6) (P>0.05 vs. control). Barkor/ATG14, Beclin1-associated autophagy-related key regulator; qRT-PCR, quantitative real-time polymerase chain reaction.

To elucidate the potential role of Barkor, Saos-2 cells were transfected with Barkor or control siRNA. The expression of Barkor was observed by western blotting and qRT-PCR. The results revealed that the downregulation of Barkor was significant (transfection efficiency >70%) (Fig. 2A). A CCK-8 assay was performed to determine the effects of the transfection of Barkor-siRNA on cell growth and death. Control, control-siRNA-transfected and Barkor-siRNA-transfected cells exhibited similar cell growth and death (Fig. 2B). Silencing of Barkor thus had no direct effect on host cell growth and apoptosis.

### Interference of Barkor-sensitized Saos-2 cells to cisplatin

**Figure 3:**
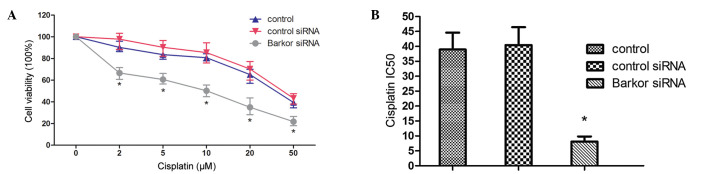
Knockdown of Barkor sensitizes Saos-2 cells to cisplatin. (A) Cytotoxicity of cisplatin in Saos-2 cells, Saos-2 Barkor-silenced cells and control-siRNA-transfected Saos-2 cells. The cells were treated with cisplatin at the indicated concentrations for 48 h. Percent survival was determined using the CCK-8 assay. (B) IC_50_ values for cisplatin in the different cells. The IC_50_ values were defined as the concentration causing 50% growth inhibition in treated cells, compared to that in control cells and were determined after 48 h of exposure to cisplatin. Bars show the average intensity of each band ± standard deviation (n=6) (^*^P<0.05). Barkor/ATG14, Beclin1-associated autophagy-related key regulator.

**Figure 4:**
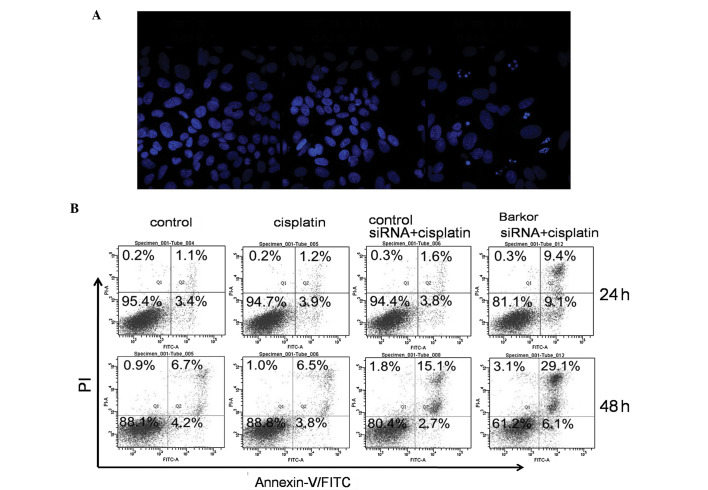
DAPI staining and Annexin-V/PI assay were used to detect apoptosis. (A) Untransfected, Barkor-siRNA- and control-siRNA-transfected Saos-2 cells were treated with cisplatin (2 μM) for 48 h and then fixed and stained with DAPI. Morphological changes were visualized by laser confocal fluorescence microscopy. Nuclear fragmentation and apoptotic bodies were clearly apparent in Barkor-siRNA-transfected cells, while no nuclear fragmentation or apoptotic bodies were evident in the control-siRNA-transfected or untransfected Saos-2 cells treated with cisplatin (2 μM). (B) Annexin-V/PI apoptosis assay of control, Barkor-siRNA- and control-siRNA-transfected cells treated with 0 and 2 μM of cisplatin for 24 and 48 h, respectively. The relative percentage of live (lower-left quadrant), early apoptotic (lower-right quadrant), late apoptotic (upper-right quadrant) and necrotic (upper-left quadrant) cells is shown. DAPI, 4′,6-diamidino-2-phenylindole; Barkor/ATG14, Beclin1-associated autophagy-related key regulator.

Knockdown of Barkor in Saos-2 cells increased their sensitivity to cisplatin. Fig. 4A shows the decrease in cell proliferation as a function of drug concentration following treatment with cisplatin for 48 h. Barkor-silenced Saos-2 cells showed evident susceptibility to cisplatin compared with the untransfected and control-siRNA-transfected parent cells. No significant difference in survival was observed between control-siRNA-transfected cells and untransfected Saos-2 cells (Fig. 3A). Results of the CCK-8 assay examination showed that after 48 h treatment, IC_50_ values for cisplatin were 37.13 μM in untransfected cells, 41.56 μM in control-siRNA-transfected cells and 7.86 μM in Barkor-silenced cells (Fig. 3B).

### Apoptosis induced by cisplatin in Barkor-silenced Saos-2 cells

We quantified cisplatin-induced apoptosis of control- and siRNA-transfected Saos-2 cells. DAPI staining was used to visualize nucleosomal DNA damage. Pyknosis, crescent-shaped edge set, nuclear fragmentation and apoptotic bodies were evident in Barkor-silenced Saos-2 cells treated with cisplatin (2 μM), while these morphological features were not apparent in the control-siRNA-transfected or untransfected Saos-2 cells treated with cisplatin (2 μM) (Fig. 4A). As shown in Fig. 4B, Barkor-siRNA-transfected Saos-2 cells treated with cisplatin had much higher rates of apoptosis than the control and control-siRNA-transfected cells.

### Knockdown of Barkor and upregulation of caspase-12 combined with cisplatin treatment

**Figure 5:**
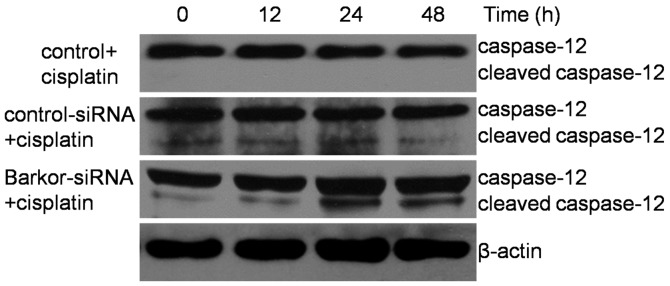
Determination of caspase-12 by western blotting following treatment of Saos-2 cells with cisplatin. Saos-2 cells were treated with cisplatin (10 μM) and the caspase-12 levels were examined over a period of 12–48 h. Cisplatin treatment induced a significant increase in caspase-12 level in Barkor-siRNA-transfected Saos-2 cells. Barkor/ATG14, Beclin1-associated autophagy-related key regulator.

Chemotherapeutic agents have previously been found to activate autophagy in osteosarcoma (18). In the present study, treatment of Saos-2 cells with cisplatin (10 μM) for 12–48 h induced an increase in the Barkor level (Fig. 1). Moreover, we investigated the effect of cisplatin on the caspase-12 protein expression, which is key in ER-mediated apoptosis. Of note, caspase-12 was not cleaved in control-siRNA-transfected Saos-2 cells but in Barkor-siRNA-transfected Saos-2 cells undergoing ER stress-induced apoptosis (Fig. 5). These findings suggest that the sensitization of cells by silencing of Barkor occurs through activation of ER-mediated apoptosis.

### Endoplasmic reticulum- and mitochondrial-mediated apoptosis induced by cisplatin in Barkor-silenced Saos-2

**Figure 6:**
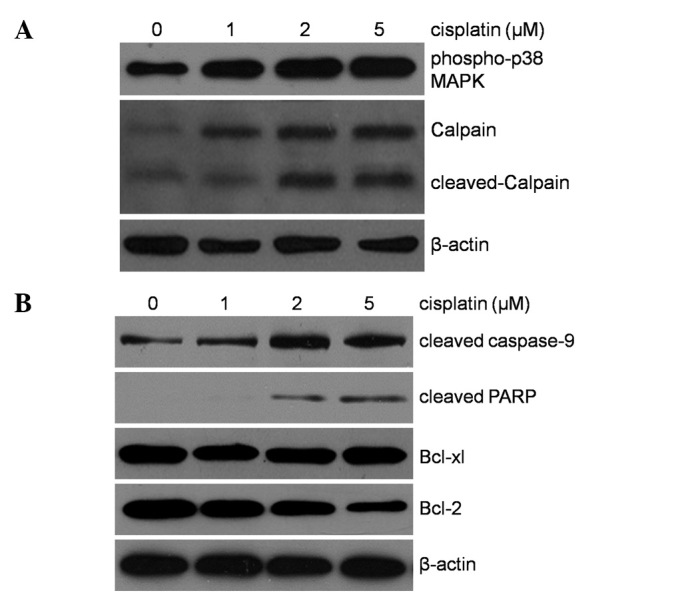
Western blot analysis of phospho-p38 MAPK, calpain, cleaved caspase-9, cleaved PARP and antiapoptotic proteins, Bcl-xL, Bcl-2 (A and B), in whole cell lysates of Barkor-siRNA-transfected Saos-2 after treatment with 0, 1, 2, 5 μM of cisplatin for 48 h. Downregulation of antiapoptotic marker Bcl-2 was evident in Barkor silenced cell lines treated with 5 μM of cisplatin. Cisplatin apparently activated the expression of phospho-p38 MAPK, calpain, cleaved caspase-9, cleaved PARP. Barkor/ATG14, Beclin1-associated autophagy-related key regulator.

To determine whether apoptosis induction by Barkor knockdown and cisplatin treatment is ER- or mitochondrial-related, western blotting was used to detect phospho-p38 MAPK, calpain, PARP, Bcl-2 and Bcl-xl levels in Saos-2 cells treated with Barkor knockdown and/or cisplatin treatment (1, 2 and 5 μM). As shown in Fig. 6A, significantly detectable activation of phospho-p38 MAPK, caspase-12 and cleaved calpain suggested ER-mediated apoptosis was initiated. Fig. 6B shows that the upregulation of cleaved caspase-9 and cleaved PARP expression was observed in Barkor-transfected-Saos-2 cells treated with cisplatin, whereas Bcl-2 was markedly downregulated in dose-dependent cisplatin-treated Barkor-transfected-Saos-2 cells. However, cisplatin had little effect on the expression of another mitochondrial-mediated apoptosis marker, Bcl-xl. These results indicated that Silencing of Barkor/ATG14 sensitizes cisplatin-resistant osteosarcoma cells to ER stress- and mitochondrial-associated apoptosis.

## Discussion

Osteosarcoma is an aggressive neoplasm representing the most common primary malignant bone tumor (19). The survival rate of patients with osteosarcoma has increased as a result of rapid advancements of comprehensive therapy, particularly adjuvant chemotherapy. Nevertheless, the effect of cytotoxic drugs on osteosarcoma becomes less useful due to acquired chemoresistance. The specific drug-resistant mechanism and molecular target should be explored to overcome resistance to chemotherapeutic drugs.

Autophagy, an intracellular catabolic process, has been shown to play a critical role in multiple biological functions, including mitochondrial turnover, neuronal function, protein degradation, cell survival, and energy metabolism (20). The molecular machinery of autophagy has been identified in yeast and is referred to as autophagy-related (ATG) genes, one of which ATG14 (also known as Barkor for Beclin 1-associated autophagy-related key regulator) (21,22). Barkor is part of a protein complex comprising Beclin 1, Vps15, and Vps34, and this Atg14-containing complex is crucial in the initiation process of autophagy (21,22). Results of recent studies have shown that autophagy may help cancer cells to survive in response to growth-limiting conditions such as the presence of anticancer drugs (8,9,18). In the present study, the hypothesis that silencing of Barkor/ATG14 sensitizes cisplatin-resistant osteosarcoma cells to ER stress-associated apoptosis was tested and verified.

In the present study, the results of the experiments of molecular biology were based on the assumption of a cell survival rate of >80%. As a consequence, the effect of Barkor knockdown on osteosarcoma cell-enhanced apoptosis is probably not due to toxicity of transfection reagent and plasmid. Barkor is an evolutionarily conserved protein that exists in a variety of organisms of yeast and mammals. The Atg14 protein has two indispensable domain structures: the coiled-coil domain (CCD) in the N-terminal region and the Barkor autophagosome targeting sequence (BATS) domain in the C terminus (10,21,23). The coiled-coil domain is responsible for the interaction with Beclin 1, and the BATS domain is required for Atg14 targeting to an autophagosome. The critical roles of Barkor in autophagy have been firmly established in cell-based systems or lower organisms (10,11,21,23). However, its gene regulation and physiological functions in cancer are unclear. Findings of the present study showed that Atg14 is significantly upregulated in cisplatin-treated the Saos-2 osteosarcoma cell line. Of note, activation of caspase-12 and calpain was detected in Barkor-inhibited Saos-2 cells, but not in untransfected and control-siRNA-transfected Saos-2 cells. These results suggest that Barkor regulates the ER stress-induced apoptosis-related genes calpain and caspase-12.

To determine whether apoptosis induction by Barkor knockdown and cisplatin treatment is ER stress-mediated, western blotting, Annexin V-PI apoptosis assay and DAPI staining were used to detect apoptosis in Saos-2 cells treated with Barkor knockdown and/or cisplatin treatment. The results have shown that the protein level of caspase-12 and calpain in cisplatin-induced Barkor-silenced Saos-2 cells was significantly increased. Additionally, apoptosis induction by Barkor knockdown and cisplatin treatment is associated with ER stress and mitochondria.

In conclusion, findings of the present study have demonstrated that Barkor as a critical regulator of autophagy and protective molecule induced the cisplatin resistance of human osteosarcoma cells *in vitro*. Inhibition of this survival response is an effective method for chemosensitization of this malignant osteosarcoma. The results of this study suggest that knockdown of the Barkor expression may have clinical therapeutic applications in enhancing the efficacy of cisplatin in osteosarcoma.

## Abbreviations

Barkor/ATG14: Beclin1-associated autophagy-related key regulator
DAPI: 4′,6-diamidino-2-phenylindole
ATG: autophagy-related genes
ER: endoplasmic reticulum
qRT-PCR: quantitative real-time polymerase chain reaction
BATS: Barkor autophagosome targeting sequence
CCD: coiled-coil domain
